# Tricuspid stenosis due to pectus excavatum in a paediatric patient with trisomy 21

**DOI:** 10.1093/ehjcr/ytae104

**Published:** 2024-02-19

**Authors:** Yuya Fujita, Ayako Chida-Nagai, Atsuhito Takeda

**Affiliations:** Department of Pediatrics, Hokkaido University, Kita 15, Nishi 7, Sapporo 060-8638, Hokkaido, Japan; Department of Pediatrics, Self Defense Forces Sapporo Hospital, Hokkaido, Sapporo, Japan; Department of Pediatrics, Hokkaido University, Kita 15, Nishi 7, Sapporo 060-8638, Hokkaido, Japan; Department of Pediatrics, Hokkaido University, Kita 15, Nishi 7, Sapporo 060-8638, Hokkaido, Japan

A male infant was diagnosed with trisomy 21 (T21) and complete atrioventricular septal defect at birth. Pulmonary artery banding was performed at 3 months, followed by intracardiac repair at 5 months. Transthoracic echocardiography (TTE) after the second surgery noted a tricuspid valve (TV) annulus diameter of 12.2 mm [105% of normal TV (%*N*)], with a monophasic TV inflow of 120 cm/s.

Throughout the outpatient follow-up, the pectus excavatum (PE)-related chest deformity progressed. At 3 years old, TTE revealed mild tricuspid stenosis (TS) with a mean transvalvular pressure gradient of 4.5 mmHg. The maximum TV inflow exhibited an E-wave of 113 cm/s and an A-wave of 167 cm/s (*Panel A*, Supplementary material online, *Video S1*), indicating right ventricular diastolic dysfunction. Tricuspid valve diameter was 8.8 mm (52%*N*). Contrast-enhanced chest computed tomography displayed cardiac displacement and right heart compression due to PE (*Panel B*). The Haller index (calculated by dividing the transverse diameter of the chest by the anteroposterior diameter) was markedly elevated, measuring 6.1. In *Panel C*, narrowing of the TV can be observed (arrow), as indicated. Concerning the worsening chest deformity and TS progression, PE surgery was performed. Post-operative TTE displayed a mean transvalvular pressure gradient of 2 mmHg, E-wave of 95 cm/s, and A-wave of 122 cm/s (*Panel D*, Supplementary material online, *Video S2*). The annulus diameter measured 17.0 mm (99%*N*), suggesting TS improvement.

A previous report documented instances of right ventricular obstruction resulting from PE, but our report represents the first case of pure TS due to PE in a paediatric patient with T21 after congenital heart disease surgery. We postulate severe PE development from T21-related joint hypermobility compounded by cardiac surgery. In cases of worsening PE, thorough cardiovascular evaluation, including TTE and chest computed tomography, is vital. In conditions such as T21-associated joint hypermobility, remaining vigilant for the possible development or worsening of PE following cardiac surgery is imperative.

**Figure:**
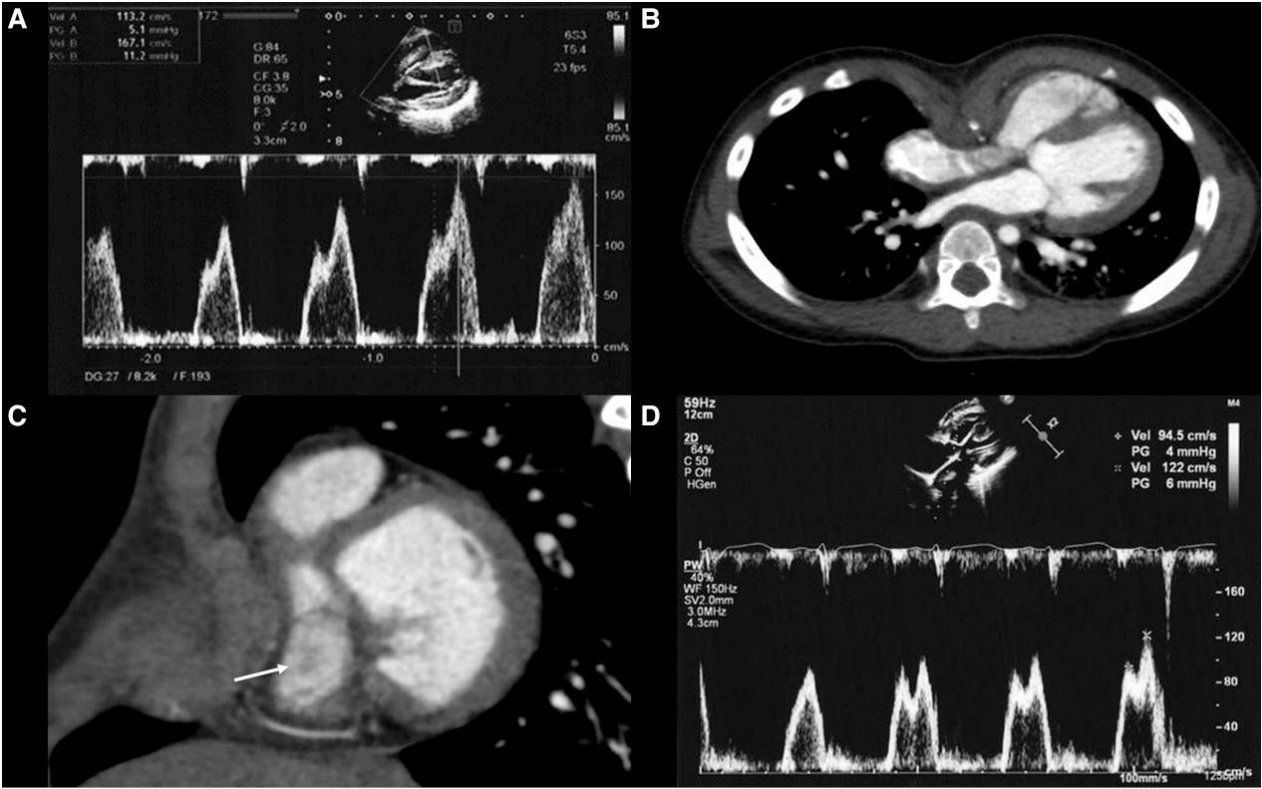
(*A*) Pulsed Doppler image of tricuspid valve inflow blood flow before pectus excavatum surgery. (*B*) Horizontal sectional image of chest contrast-enhanced computed tomography before pectus excavatum surgery. The Haller index was 6.1. (*C*) Chest contrast-enhanced computed tomography image of the cross-sectional view looking down at the tricuspid valve (arrow) from the right atrium side before pectus excavatum surgery. (*D*) Pulsed Doppler image of tricuspid valve inflow blood flow after pectus excavatum surgery.

## Supplementary Material

ytae104_Supplementary_Data

## Data Availability

The data underlying this article will be shared on reasonable request to the corresponding author.

## Supplementary material

**Consent:** Written informed consent was obtained from the guardian of the patient in accordance with the Committee on Publication Ethics (COPE) guidelines.

**Funding:** None declared.

